# Binary Neural Network for Automated Visual Surface Defect Detection

**DOI:** 10.3390/s21206868

**Published:** 2021-10-16

**Authors:** Wenzhe Liu, Jiehua Zhang, Zhuo Su, Zhongzhu Zhou, Li Liu

**Affiliations:** 1 College of Systems Engineering, National University of Defense Technology, Changsha 410073, China; liuwenzhe15@nudt.edu.cn; 2 Center for Machine Vision and Signal Analysis, University of Oulu, 90570 Oulu, Finland; Jiehua.Zhang@oulu.fi (J.Z.); Zhuo.Su@oulu.fi (Z.S.); 3 School of Computer Science and Engineering, Sun Yat-sen University, Guangzhou 510006, China; zhouzhzh8@mail2.sysu.edu.cn

**Keywords:** automated defect detection, binary network, binary neural network, efficient network, automated visual inspection, surface defect detection

## Abstract

As is well-known, defects precisely affect the lives and functions of the machines in which they occur, and even cause potentially catastrophic casualties. Therefore, quality assessment before mounting is an indispensable requirement for factories. Apart from the recognition accuracy, current networks suffer from excessive computing complexity, making it of great difficulty to deploy in the manufacturing process. To address these issues, this paper introduces binary networks into the area of surface defect detection for the first time, for the reason that binary networks prohibitively constrain weight and activation to +1 and −1. The proposed Bi-ShuffleNet and U-BiNet utilize binary convolution layers and activations in low bitwidth, in order to reach comparable performances while incurring much less computational cost. Extensive experiments are conducted on real-life NEU and Magnetic Tile datasets, revealing the least OPs required and little accuracy decline. When classifying the defects, Bi-ShuffleNet yields comparable results to counterpart networks, with at least 2× inference complexity reduction. Defect segmentation results indicate similar observations. Some network design rules in defect detection and binary networks are also summarized in this paper.

## 1. Introduction

Product defects in production or periodic maintenance [1] are fairly common; however, any quality problems in products can cause hidden dangers for life and property, and further adverse impacts in sales and reputation to enterprises, especially in numerous areas of precise instruments and national industries; for example, aerospace, civil transportation and infrastructure, and machinery engineering. Naturally, for the reason that defects pose a serious threat to the durability and quality of products, defect inspection becomes a key component in both production and maintenance processes. Generally, surface defect detection is used to guarantee that products are visually free of irregularities or defects on the surface, which is also known as non-destructive testing [2], automated optical inspection [3], or optical quality control [4]. Recently, in both industry and academia, people are paying more attention to the necessity of adequate and careful inspection.

Previously, defect detection was achieved by skilled inspectors, which resulted in high cost and low efficiency [5]. On the one hand, human-based visual inspection requires intensive training of laborers, but is still subjective and time-consuming [6], and it can sometimes be dangerous to perform manual onsite inspection [6]. On the other hand, experienced workers with long-term continuous work will suffer from vision fatigue, leading to an inevitable accuracy degradation. In light of this, increasing demand for both quality assurance and industrial automation is gradually being satisfied by deep learning and machine vision technology [3], with low cost, human labor relief, high efficiency, and high reliability. For example, Cognex ViDi Suite [7], a commercial ready-to-use piece of software, embeds Red Tool dedicated to irregularity detection, aesthetic visual inspection, and saliency segmentation.

**Figure 1:**
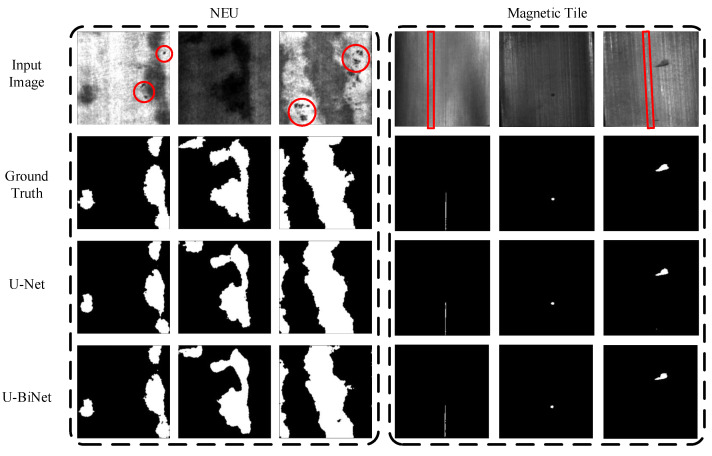
From top to bottom: input images; pixel-wise annotation of corresponding input image; prediction of full-precision U-Net and prediction of U-BiNet in binary.

The difficulties in visual defect detection mainly lie in two parts: characteristics of defect and model efficiency. Initially, due to the complex production environment, images may contain various noise [5] caused by high temperature [8], dense mist [3], uneven illuminations [9,10,11,12], aperiodic vibration [3], and other factors. For example, dark spots (samples are surrounded by red circles) in the first and third columns in Figure 1, and lighter vertical scratches (samples are framed by red rectangles) in the fourth column and the column on the far right. As for the defect itself, an inspection may suffer from its tiny scale (three columns on the right in Figure 1), random distribution (occasionally touching the boundaries of the images, as shown in the first and fourth columns in Figure 1), background clustering (due to various and complex surface textures, and low background–foreground contrast [5], as shown in the second columns of both datasets in Figure 1).

Despite the above difficulties, another urgent demand in the next phase of manufacturing, namely Industry 4.0 [13], is the efficiency of methods, mainly including requirements on the storage and inference speed of the model [5]. As for the inference time, in most of the production phase, the highly mechanized assembly line requires defect detection to be in strict real-time. Taking steel production as an example, its high-level time cost requirement is caused by the rapid casting and rolling speed of steel slabs in real-life manufacturing shops. Therefore, the speed of detection is a constant pursuit of researchers; as stated in [14], an SVM classifier can take 0.239 s on CPU to recognize defects in a single defect photograph when testing. In contrast, for convolutional detection networks, imperfections can be detected in milliseconds with truthful location and scale information of defects. For example, the YOLO-based method [14] takes only 0.012 s to process a raw defect photograph. All of these contribute to performance-sufficient and cost-efficient models that can be deployed to realize strict requirements of speed. Despite this, there have been few tentative attempts to investigate the network redundancy and efficiency in defect detection.

To remedy this, our main contributions are summarized as follows:

- First, to the best of our knowledge, this paper provides the first exploration of a binary network on defect detection tasks;
- Second, we select powerful and even more compact backbones, apply ReActNet in the application of surface defect detection, and propose U-BiNet and Bi-ShuffleNet to further improve the efficiency in defect segmentation and classification, respectively.
- Third, we conduct adequate experiments on on-the-spot datasets, in which we considerably reduce the inference time and computational cost for defect detection, while remaining faithful and providing promising results.

## 2. Related Work

### 2.1. Surface Defect Detection

Surface defect detection is used to ensure the proper quality of a finished product. In rapidly developing modern industry, the detection procedure can be exceedingly repetitive and exhausting; thus, computer vision methods have dominated in the automatic quality assessment of diverse industrial products, covering bolts [15], fasteners [16], LED chips [17], etc.

Generally, surface defect detection tasks can be coarse-grained or fine-grained based on the different industrial requirements. Coarse-grained detection [8,18,19,20] aims to identify whether there are any defects or not, and the exact defect type, which is closely related to classification in machine vision; whereas fine-grained [9] detection aims to recognize defective regions from a normal background and locate the defect in a pixel-wise or bounding-box manner, which is more likely to be simplified object detection and segmentation.

In terms of technology in defect detection, we can roughly classify prior work into traditional methods and deep-learning-based methods. For most traditional methods, they are mainly based on gray-scale value, gradient edge, and handcrafted optical features on images; well-known methods include wavelet [10], curvelet [2], and shearlet [21,22] transformation. For example, Li et al. [23] employed threshold-based approaches with the assumption that crack pixels are usually darker than their neighbors. However, such intensity-based methods are no longer appropriate when handling surfaces with strong or complex textures or noise. Song et al. [8] proposed AECLBPs and introduced an adjacent evaluation window around the window to modify the threshold scheme of the CLBP, showing robustness to additive Gaussian noise. However, these methods require the specification of expert rules, which is only suitable in a particular domain, but may fail when applied to a new problem set, for the reason that every problem varies in its distinctive features, only responding to a specific feature extractor. According to the Industry 4.0 paradigm and a wide variety in surface defect categories [5], the tendency is switching towards flexibility in manufacturing along with higher generalization, where quick transfer to a different defect is vital [24].

Recently, as a more unified method, deep learning methods, especially CNN [25], have become dominant in the defect detection area and accomplish state-of-the-art performance [19,20], with a minimum of human interference or expertise. Masci et al. [19] demonstrated that for defect classification of surface photographs, a deep-learning-based method can exceed classical machine-vision methods, where hand-crafted features are usually merged with support vector machines. However, as these neural networks did not apply advanced ReLU and batch normalization, their structures are limited to five-layer and shallow. Then, Faghih et al. [26] embedded ReLU as the activation function and compared networks in various depths for rail imperfection recognition. As the depth of CNN increases, on the one hand, it can obtain much higher performance: Cha et al. [18] applied a CNN to recognize defects on concrete and steel, achieving approximately 98% accuracy, which shows great robustness in extensively varying real-world situations; on the other hand, massive computational consumption is behind the stronger results, leading to redundant parameters, complex calculation, and further, deployment difficulty. In pursuit of industrial application in real-time scenes, efficient networks are introduced into defect detection tasks. For example, Cha et al. [27] applied a faster R-CNN model; Li et al. [14] modified the YOLO network.

In summary, though extensive research has explored effective defect detection models, after rounds of technology iteration, as pointed out by Luo et al. [3], a crucial challenge at present can be a better tradeoff of detection accuracy and computing efficiency. Though neural networks have considerably reduced the average processing time for each image compared with traditional methods, neural networks can still be prohibitively energy-intensive and relatively time-consuming, which is far from meeting the standard of real-time and portal device deployment. Thus, light-weight and fast models are required for broader applicability, e.g., embedded systems and mobile devices. However, few attempts have been made to consider defect inspection in constrained environments.

### 2.2. Binary Network

Currently, not limited to the field of defect detection as discussed above, a state-of-the-art DCNN usually has many parameters and high computational complexity, which both impede its application in hand-held devices and slow down the iteration of its research and development. In light of this, researchers have made vast inroads into network compression and acceleration. Representative technologies include network pruning [28], neural network search [29], quantization [30], etc.

Empirically, real-valued parameters are not necessary when achieving high performance in DCNNs. To this end, network quantization is proposed to reduce both model size and computational burden by using low-bitwidth weights and low-bitwidth activations. A binary network, or 1-bit CNN, is the extreme scenario of a highly quantized network with the maximum compression ratio of 1 bit. In detail, the 1-bit weight and activation are obtained by means of a sign function,

(1) $$x^{b}=Sign\left(x\right)=\left\{\begin{array}{cc} +1 & if\;x\geq 0,\\ -1 & otherwise, \end{array}\right.$$

where *x* is the real-valued variable and $x^{b}$ is the binarized variable (weight or activation), existing in both the training and inference phases of 1-bit CNN.

In the latest work, weights and activations keep the binary at run-time when computing the variable gradients during training. Compared to the normal full-precision DCNN with a 32-bit weight parameter, a binary network enjoys up to 32× memory saving, in which computationally prohibitive matrix multiplication operations also become cost-efficient bitwise XNOR operations and bit-counting (accordingly, up to 64× computation saving [31]). As the pioneering work, both BinaryConnect [32] and BinaryNet [33] achieve comparable accuracy as real-value CNNs on MNIST and CIFAR-10. The subsequent ReActNet [34] reduced the top-1 accuracy gap to a full-precision counterpart to at most 3.0% on the ImageNet dataset while realizing considerable memory saving and inference acceleration. Thus, binary CNN is an effective method to balance the contradiction between descriptive power and computational complexity. To this end, binary networks have been widely applied from basic classification [31,34] to some more advanced applications, e.g., single-image super-resolution [35] and object detection [36].

## 3. The Proposed Methods

### 3.1. Bi-ShuffleNet

Currently, well-known binary networks for feature extraction and classification are usually on the basis of Resnet [31] and MobileNet [34]. Further compressing more compact networks would be more convincing and of greater concern for practical application, thus, we chose ShuffleNet V2 (0.5×) as our binarization backbone, instead of other non-compact structures. From the practical point of view, ShuffleNet V2 (0.5×) [37] has fewer FLOPs, memory usage, and parameters, and faster inference time than MobileNet.

For the baseline model, we initially adopted 1-bit convolutions to replace all the convolution layers in ShuffleNet V2 (0.5×), except the first and the last convolution layers in the network and outside the stacking units, which remained in full precision instead. We also applied ReAct operations (i.e., Rsign and RPReLU) proposed in [34] to activation binarization and activation function design, which provide channel-wise shifting and reshaping capacity on the distribution of the activations to learn more representation, simply by adding small learnable variations to activation distribution with little computational burden addition. The effect of these subtle shifts is significant because binary activations are much more sensitive to these small values, mostly leading to completely different results; in contrast, real-valued activations are robust because the detailed information will be maintained largely in full precision [34]. As illustrated in [34], the 1-bit activation is achieved by

(2) $$a_{i}^{b}=RSign\left(a_{i}\right)=\left\{\begin{array}{cc} +1 & if\;a_{i}\geq \gamma_{i},\\ -1 & otherwise, \end{array}\right.$$

where $a_{i}$ is the real-valued activation on the *i*-th channel and $a_{i}^{b}$ is the binarized output after RSign, existing in both training and inference stages of the 1-bit CNN. $\gamma_{i}$ is a learnable coefficient for activation on the *i*-th channel, controlling the threshold.

In [34], RPReLU is defined as

(3) $$RPReLU\left(a_{i}\right)=\left\{\begin{array}{cc} a_{i}-\zeta_{i}+\eta_{i} & if\;a_{i}\geq \zeta_{i},\\ \beta_{i}\left(a_{i}-\zeta_{i}\right)+\eta_{i} & otherwise, \end{array}\right.$$

where $a_{i}$ is the real-valued activation on the *i*-th channel, and $\zeta_{i}$ and $\eta_{i}$ are learnable shifts for distributional reshaping. $\beta_{i}$ is also a learnable coefficient in the original PReLU, in order to control the slope of the left side. Similar to RSign, all the coefficients are permitted to vary across channels.

**Figure 2:**
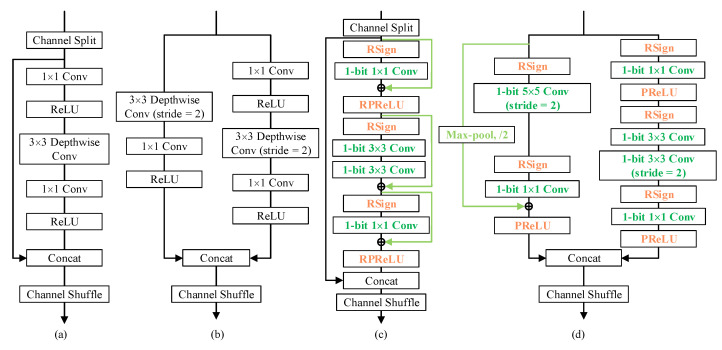
Building units of ShuffleNet V2 (**a**,**b**) and Bi-ShuffleNet (**c**,**d**). For the sake of brevity, the batch normalization layer after each convolution layer is omitted.

In light of the characteristics of defect detection tasks, the 3 × 3 depth-wise convolutional layers in the right branch of both basic unit (Figure 2a) and spatial down-sampling (2×) unit (Figure 2b) are replaced by 2 consecutive 1-bit 3 × 3 convolutions while the left branch of down-sampling unit (Figure 2b) is substituted by 1-bit 5 × 5 convolutions, in order to enjoy a larger receptive field to detect defects of various scales. Applying multiple 3 × 3 convolutions in sequence to enjoy a larger reception field is a common and efficient idea in object segmentation [38,39,40] to save parameters in the meantime. Correspondingly, the kernel size of the first convolution layer is defined to be 9 × 9 as well.

To sufficiently reuse the real-valued activation, which has proven to be crucial for the accuracy [34], we employed parameter-free identity shortcuts to bypass all the intermediate convolution layers in the basic unit (Figure 2c) and connected the input by skipping two convolutional layers at the left branch in the spatial down-sampling unit (Figure 2d), with near-zero computational cost addition. In terms of spatial mismatch of shortcuts in spatial downsampling units, we used max pooling to ensure small but important details survive in downsampling, which is extremely beneficial in defect detection tasks.

Lastly, after incorporating all these ideas above, an efficient channel shuffle was then applied to encourage information exchange across the two branches. After the shuffling, the next block starts by repeatedly stacking. Thus, the proposed building blocks, as shown in Figure 2c,d, as well as the built structures, are named Bi-ShuffleNet. We showed that the proposed Bi-ShuffleNet achieves comparable or even more superior performance than existing binary networks with an even lower computational budget.

### 3.2. U-BiNet

**Figure 3:**
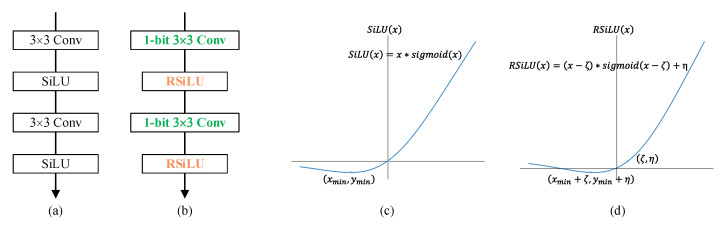
(**a**) Building blocks of U-Net; (**b**) basic unit for proposed U-BiNet; (**c**) SiLU activation function; and (**d**) proposed RSiLU activation function with learnable coefficients.

In terms of the fine-grained defect segmentation task, we chose the powerful U-Net [41] as the backbone, whose basic unit is shown in Figure 3a. U-Net was initially proposed to address problems in the biomedical field, but rapidly developed to other segmentation areas, due to its strength to re-use multi-scale intermediate feature maps, which is also of great significance in saliency detection tasks. In order to reduce parameters and computational consumption, the output channel of each convolution in the U-Net structure, no matter whether in full precision or binary, in this paper is half of that in the original U-Net [41], with negligible performance degradation. Similar to the construction of Bi-ShuffleNet, we initially replaced all vanilla convolutional layers with binary convolutional layers. However, for the reason that richer semantic feature representation required by pixel-wise prediction tasks cannot be fully achieved by a 1-bit CNN, we still kept the intermediate activations as full-precision, and tried to reduce the bitwidth of activations to as low as possible, by means of

(4) $$a^{q}=\frac{round(\left(2^{k}-1\right)a)}{2^{k}-1}$$

where full-precision activations *a* are quantized to $a^{q}$ in *k*-bit.

In this way, the inference complexity is still improved because multiplication turns into an efficient addition-subtraction operation, though the increment is certainly less than that of complete 1-bit CNN. However, its saved model size is the same as 1-bit CNN.

Equally with the motivation of RPReLU, in order to enable explicit learning of the distribution shape in real-valued networks, we actually modified the SiLU [42], which is also known as Swish,

(5) $$SiLU\left(a\right)=a*sigmoid\left(a\right)=a\frac{1}{1+e^{-a}}$$

to RSiLU, with more flexibility to adaptively learn parameters for distributional reshaping,

(6) $$RSiLU\left(a_{i}\right)=\left(a_{i}-\zeta_{i}\right)*sigmoid\left(a_{i}-\zeta_{i}\right)+\eta_{i}=\left(a_{i}-\zeta_{i}\right)\frac{1}{1+e^{-a_{i}+\zeta_{i}}}+\eta_{i}$$

where $a_{i}$ is the activation on the *i*-th channel, and both $\zeta_{i}$ and $\eta_{i}$ are learnable shifts for distributional reshape. Similar to RPReLU, all the coefficients vary across channels. The shape comparison between SiLU and RSiLU is shown in Figure 3c,d.

Therefrom, the basic building unit is as shown in Figure 3b. The further constructed structure still efficiently utilizes multi-scale features and remains U-shaped, and is named U-BiNet.

## 4. Experiments

### 4.1. Datasets

In order to verify the superiority of the proposed model, we utilized real-world examples in two datasets below, instead of using synthetic ones.

Magnetic Tile (MT) [9] provides 472 surface defect images, with 6 classes for classification. Due to the scarcity of the data, we augmented the randomly split training set by rotation (0°, 90°, 180°, and 270°) and horizontal flipping, leading to a training set 8× larger than the unaugmented training set. Though most of the images in MT contain a certain type of rain-streak-like noise and severe vignette effect in the corner, we still gave up any crop operation, which was applied in [11] to ensure the most relevant defective regions exist in patches.

The NEU Surface Defect Database [43] is composed of 300 photographs per class and 6 classes (rolled-in scale, patches, crazing, pitted surface, inclusion, scratches) in total with defects whose size is 200 × 200 for classification; for segmentation, [43] do not provide pixel-wise labels, but bounding box annotations. Only for patch defects did we obtain pixel-wise ground truth from [11,12]. For patch defects, there were roughly 22.9% of pixels labeled as defects, while the other 77.1% were labeled as non-defects. Messy backgrounds with a low signal-to-noise ratio (SNR) make it a more challenging task.

There is no formal data split for training, validation, and test sets in either dataset; therefore, we randomly partitioned them in a 7:1:2 fashion. All the performance results reported in the paper were calculated in 5 independent splits on average for credibility, if not specified.

### 4.2. Experimental Details

The training was conducted on the training set initially, and the trained parameters with the highest validation accuracy across all iterations were adopted for testing. All the binary networks were trained in two rounds because of RSign, RPReLU, and RSiLU in proposed networks, as in [34]. At the first stage, we trained the learnable variables of RSign, RPReLU, or RSiLU with real-valued weight parameters from scratch. Then, at the second stage, binary networks were initialized by the weights learnt in the first stage, and fine-tuned with weights in the binary version. It is worth noting that, at both stages, the backpropagation was guided by cross-entropy loss between the binary network output and the ground truth. Other details are given below.

Coarse-grained task: In terms of Magnetic Tile, for all real-valued networks, the batch size was set as 32, training for 200 epochs with the Adam optimizer; the loss calculation is based on Tversky Loss [44], which is widely applied in defect detection and lesion attribute segmentation, due to its strength in data imbalances. The initial learning rate was $10^{-5}$, and was adjusted by the One Circle method [45] during training.

As for NEU, for transferred Resnet18, Resnet34, and MobileNet V2, we employed the Adam optimizer and set the learning rate as $10^{-3}$ when training on the target dataset. For the real version of ReActNet and BiRealNet, we also applied the Adam optimizer, but set the learning rate to $10^{-5}$. All the hyperparameters above were selected by both experience and grid search to avoid the training process falling into under-fitted or over-fitted situations.

In terms of all the 1-bit CNNs, the learning rate was decayed with the cosine annealing strategy and warm-up was applied for the first 5 epochs. The initial learning rates were $10^{-4}$ and $5\times 10^{-4}$, respectively, in step 1 and step 2. In the first round of 1-bit CNN training, only learnable parameters in RSign and the activation function are optimized in priority, while both parameters and weights in 1-bit convolutions are optimized together in the second round, thus, a larger learning rate is required to accelerate. The Adam optimizer was also selected as it can normally prevent the training of 1-bit CNNs falling into the situation of local minima [46], compared with other optimizers.

Fine-grained task: For the unaugmented NEU dataset, images were rotated by a random degree in [0°, 90°, 180°, 270°], and flipped horizontally or not at a 50/50 probability in the data pre-processing step. For the original full-precision U-Net, the batch size was set as 32, training for 200 epochs with the Adam optimizer. The learning rate was set as $10^{-4}$ and loss was calculated by the Tversky method. As for the proposed U-BiNet, at both training steps, we used the Adam optimizer for 200 epochs with batch size as 8 and learning rate as $10^{-5}$. Taking the smaller batch size and learning rate than that of U-Net into account, we intended to ensure that the training of sensitive binary parameters in U-BiNet was stable and avoid overfitting.

### 4.3. Results Analysis

#### 4.3.1. Coarse Task: Defect Classification

Experiment metrics: For classification tasks, the metrics include average recognition accuracy, standard deviations, and OPs. OPs is a rough sum of binary operations and floating-point operations, i.e., OPs = BOPs/64 + FLOPs. For the reason that inference time and computational cost are not measurable in current popular devices, which treat quantified *k*-bit values as full precision, OPs is recognized [34] to act as a proxy metric theoretically to simulate the model efficiency in the research of network binarization.

Coarse-grained Defect Detection on Magnetic Tile: Both MobileNet V2 and Resnet18 were pretrained on over 1.2 million photographs from ImageNet [47] at first and then transferred to the target dataset, whereas other models were directly trained from scratch on the target dataset. The BiRealNet reported in this paper is advanced by RSign and RPReLU with distribution reshape capacity as well. All binary networks, including BiRealNet, ReActNet, and Bi-ShuffleNet, are only supervised by the ground truth during the whole training, instead of assigning a well-trained real-valued network as the teacher model in a knowledge distillation manner, which may lead to unsatisfied feature representation, and further non-convergence problems otherwise. The OPs was calculated in the situation where a 224 × 224 RGB image was loaded into the network.

**Figure 4:**
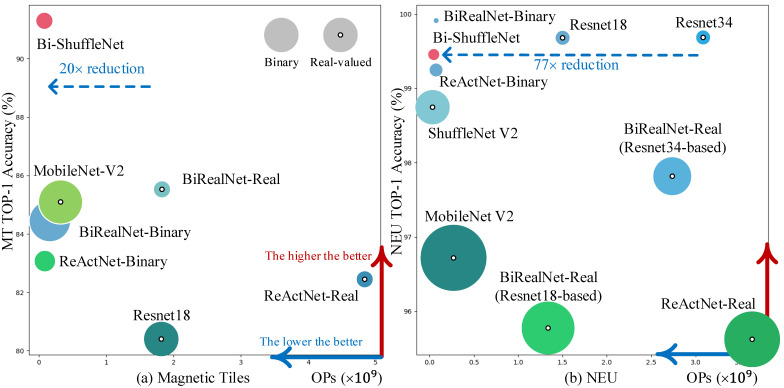
Classification performance on Magnetic Tile and NEU datasets.

Consequently, the results are displayed in Table 1, showing that the proposed Bi-ShuffleNet outperforms not only existing 1-bit CNNs, but also full-precision networks, by a large margin, in the lowest computational budget. A more intuitional demonstration is shown in Figure 4a, where the radius of the circle represents the standard derivation of the method. Therefore, the bigger the area of the circle, the less the stability of this method. With a similar binarization setting, Bi-ShuffleNet exceeds ReActNet, whose backbone is MobileNet V1, by 8.24% in accuracy, with a further 10% OPs reduction. Bi-ShuffleNet also achieves a 6.81% accuracy improvement over the BiRealNet, and enjoys the benefits of more stability (half the standard deviations of BiRealNet) and less cost (approximately 2× computational reduction).

Coarse-grained Defect Detection on NEU: We collected the results of ‘LBP+SVM’ and ‘AECLBP+SVM’ in [8]. Both MobileNet V2 and Resnet18 were pretrained on ImageNet at first and then transferred to NEU, whereas other models were directly trained from scratch. The OPs was calculated based on a 224 × 224 single-channel image.

Accordingly, the results are shown in Table 2. A more intuitional illustration is shown in Figure 4b. Generally, Bi-ShuffleNet is on par with both real-valued and binary networks in capability and stability. Specifically, our ShuffleNet-based model outperforms MobileNet-based ReActNet by 0.19% in accuracy, enjoying roughly 40% OPs reduction.

#### 4.3.2. Fined-Grained Task: Defect Segmentation

Experiment metrics: As suggested in previous work [11], we mainly employed accuracy, precision, recall, false negative rate (FNR), false positive rate (FPR), mean absolute error (MAE), and Dice similarity coefficient as performance criteria for performance evaluation.

In detail, FPR is interpreted as the percentage of defect-free pixels that are incorrectly identified as defects, and FNR measures the proportion of defective pixels that are falsely identified as non-defects. MAE is another quantitative indicator to assess the dissimilarity between the prediction and the ground truth.

Obviously, the higher the values of accuracy, precision, recall, and Dice, the lower the values of FPR, FNR, and MAE, and the closer the prediction is to human subjective feeling.

**Figure 5:**
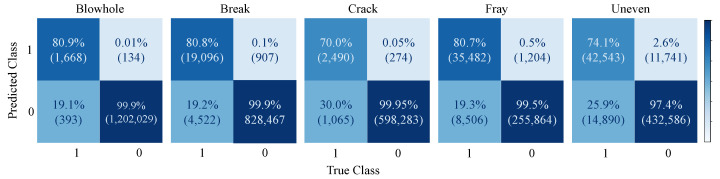
Confusion matrix of U-BiNet on the test set of Magnetic Tile. The figure is best viewed in color and zoomed in.

Fined-grained Defect Detection on Magnetic Tile: In terms of Magnetic Tile, we trained models on each defect independently to verify the robustness of our model. As shown in Table 3, U-BiNet reports results on par with the state-of-the-art in full precision. Conspicuously, with weights in binary and activations in lower bitwidth, U-BiNet demonstrates faster inference speed and less memory occupied in model saving. Alternatively, when the bitwidth of activations is controlled to be as low as 2, performances of U-BiNet show considerable degradation in metrics. A more intuitional demonstration is shown in Figure 5, where the confusion matrices of U-BiNet, whose activations are real-valued, on each defect are displayed. Accordingly, ‘crack’ and ‘uneven’ are less likely to be detected.

Fined-grained Defect Detection on NEU Patches: The results are indicated in Table 4, where the performances of CAT, SSD, and ESP are reported in [11]. For the reason that there is no recognized formula to calculate the OPs of the network whose activation in inference is quantized, we have simply listed the inference complexity introduced in [48]. Obviously, U-BiNet achieves superior performance than most existing methods, and shows a good balance among accuracy, inference complexity, and model size.

### 4.4. Ablation Study

We conducted ablation experiments to classify each component’s exclusive contribution and the collaborative contribution of each unique combination towards the overall performance.

Initially, we analyzed the individual effects of the following modifications on the binarized ShuffleNet V2 0.5× at the very beginning. The abbreviations of modifications used in this section are as below:

BL: The baseline model, where RSign and RPReLU are introduced in Shufflenet V2 0.5× while all convolutions in the repeated units are replaced with 1-bit convolutions.

$S_{basic}$: Add shortcuts in the right branch of the basic unit.

$S_{down}$: Add shortcuts in the left branch of the down-sampling unit.

$ks_{DW_{l}}$: Reset the kernel size of the 1-bit convolution layer that substitutes the depthwise convolution layer in the left branch of the down-sampling unit. In addition, $2\times ks_{DW_{l}}=3$ denotes 2 consecutive 3×3 convolution layers on the left branch to obtain the same receptive field as a single 5×5 convolution layer.

$ks_{DW_{r}}$: Reset the kernel size of the 1-bit convolution layer that substitutes the depthwise convolution layer in the right branch of both basic and down-sampling units. Similarly, $2\times ks_{DW_{r}}=3$ denotes 2 consecutive 3×3 convolution layers on right branch.

$ks_{conv1}$: Reset the kernel size of the first vanilla convolution layer in Bi-ShuffleNet.

mp: Substitute the average pooling with max pooling in the shortcut in the down-sampling unit.

Experiments were carried out on Magnetic Tile and NEU datasets, as shown in Table 5 and Table 6, respectively, where we found that those proposed modifications are independent and can contribute collectively towards improving the overall accuracy. Besides, we can also draw some conclusions, which are beneficial for the design of networks in defect detection and construction of binary networks.

As for defect detection tasks, these turn out to be more efficient when enlarging the kernel size of convolution layers, no matter whether in building units (i.e., $ks_{DW_{r}}$ and $ks_{DW_{l}}$) or ahead of basic units (i.e., $ks_{conv1}$). When extending $ks_{DW_{r}}$ to 5, the accuracy increases by 3.05% (II and III in Table 6) and 7.75% (III and IV in Table 5), respectively, in NEU and MT, without hurting stability. When $ks_{conv1}$ grows from 3 to 9, the accuracy jumps by 0.22% (III and VI in Table 6) and 10% (VI and VIII in Table 5) in NEU and MT, with comparable stability. When decomposing the 5 × 5 convolution into 2 consecutive 3 × 3 convolutions, considerable improvements can also be seen in IX, X, and XI in Table 5, which is expected as they can enhance the nonlinear representation capacity [51]. In addition, max pooling does function in downsampling when shortcuts are introduced in defect detectors. The intermediate networks with max pooling in shortcuts witness a growth of 0.95% (III and IV in Table 6) and 2.25% (V and VI in Table 5) in NEU and MT, and at most a 2× stability enhancement.

In terms of the design of a 1-bit CNN, real-valued shortcuts are of great importance for the contribution to the final accuracy. Intrinsically, a shortcut inspires the potential of the deep network by avoiding accuracy degradation [52]. Besides, as shown in Figure 2c,d, the shortcut normally connects the previous real-valued activations after activation functions to the later output of binary convolution; thus, it preserves the intermediate real-valued activations as much as possible, facilitating the network to approach the representation of networks in full precision, which is difficult and constantly pursued in the field of network binarization [53,54]. As verified in the tables, after adding shortcuts in basic and down-sampling units, the network beat the baselines by an obvious margin of 4.45% (I and II in Table 6) and 15.72% (I and III in Table 5) in NEU and MT datasets, respectively, with better stability and negligible extra computational cost.

The effectiveness of RSiLU in U-BiNet is also demonstrated in Table 7. In NEU Patches, U-BiNet with RSiLU shows overwhelming performance in each indicator. However, in MT, U-BiNet without RSiLU possesses relatively high FNR and MAE, but lower FPR, which demonstrates that it is prone to producing pseudo-results. Therefore, a logical deduction is that the addition of learnable variables on binary activations to explicitly shift activation distribution is simple yet helpful.

## 5. Discussion

On the whole, the binary models proposed in this paper have the advantages of deep learning to reduce human involvement, promote the development of intelligent manufacturing, and accelerate the landing of Industry 4.0 [13]. In comparison with previous 1-bit CNNs or even classic real-valued networks, the purpose-built 1-bit CNNs in this paper show comparable or even overwhelming performances in surface defect detection. Empirically, with the increasing scale of defect datasets, the performance of our networks can even be further improved. Compared with prior methods based on handcrafted features, our methods are data-driven with salutary robustness to various defects; among learning-based approaches, our binary models reduce inference time and save model size without compromising the performance. Facilitated by binary networks, automated inspection systems can be deployed in every corner of working shops and embedded in edge devices or other carriers to execute quality assessments with acceptable power consumption.

However, there still exist some defects that both U-Net and U-BiNet fail to detect, e.g., crack and uneven, especially when the defects are too small and take little proportion in the dataset, or the defect itself shows little dissimilarity to the background texture. Further, our binary models should be examined in broader industrial scenarios where there exist richer defects in assembly, geometry, etc. However, this assumption is currently impeded by the lack of relevant datasets.

In addition, as the bitwidth of activations in U-BiNet can still be higher than 2, there is still plenty of reduction space in computational complexity for object segmentation tasks. Incidentally, the quantitative indicators of the efficiency in neural network quantization can be further explored to obtain a quantified comparison with networks before quantization.

Additionally, the research trends of both defect detection and network binarization have gradually switched from theoretical study to on-site application. For further investigations in the future, experiments on specific hardware and production lines should be carried out for direct inference complexity measurement instead of proxy metrics and more on-site images with noise, respectively, contributing to a broader range of benefits in both industry and academia.

## 6. Conclusions

In this paper, we showed the potential of the binary network in industrial applications. Firstly, we binarized a classification network by means of ReActNet and proposed Bi-ShuffleNet, a new binary network based on a compact backbone, which is the first exploration of a binary network in defect detection, leading to an efficient defect perception. Secondly, we introduced a customized binary network named U-BiNet for defect segmentation, demonstrating the effectiveness and striking computational saving. Lastly, we conducted extensive experiments on the NEU and Magnetic Tile datasets, and found some inspiration for both accurate and robust defect detection.

## Figures and Tables

**Table 1:** Coarse-grained defect detection performance on Magnetic Tile.

Method	TOP-1	BOPs	FLOPs	OPs
	Accuracy (%)	($\times 10^{9}$)	($\times 10^{8}$)	($\times 10^{9}$)
MobileNet V2	85.10 (±6.08)	-	3.20	0.32
Resnet18	80.41 (±4.72)	-	18.19	1.82
ReActNet-A (real, MobileNet V1-based)	82.45 (±2.18)	-	48.38	4.84
BiRealNet (real, ResNet18-based)	85.51 (±2.18)	-	18.19	1.82
ReActNet-A (binary, MobileNet V1-based)	83.06 (±2.78)	4.81	0.27	0.10
BiRealNet (binary, ResNet18-based)	84.49 (±5.71)	1.68	1.45	0.17
Bi-ShuffleNet	91.30 (±2.38)	0.22	0.87	0.09

**Table 2:** Coarse-grained defect detection performance on NEU.

Method	TOP-1	BOPs	FLOPs	OPs
	Accuracy (%)	($\times 10^{9}$)	($\times 10^{8}$)	($\times 10^{9}$)
LBP + SVM	97.93 (±0.66)	-	-	-
AECLBP + SVM	98.93 (±0.63)	-	-	-
MobileNet V2	96.72 (±1.81)	-	2.68	0.27
Resnet18	99.69 (±0.38)	-	14.97	1.50
Resnet34	99.69 (±0.38)	-	30.81	3.08
ShuffleNet V2	98.75 (±0.94)	-	0.33	0.03
ReActNet-A (real, MobileNet V1-based)	95.63 (±1.53)	-	36.41	3.64
BiRealNet (real, Resnet18-based)	95.78 (±1.45)	-	13.36	1.34
BiRealNet (real, Resnet34-based)	97.81 (±1.04)	-	27.40	2.74
ReActNet-A (binary, MobileNet V1-based)	99.26 (±0.35)	3.64	0.16	0.07
BiRealNet (binary, Resnet18-based)	99.91 (±0.13)	1.29	0.53	0.07
Bi-ShuffleNet	99.45 (±0.30)	0.22	0.38	0.04

**Table 3:** Fined-grained defect detection performance on Magnetic Tile (in percentage). ‘Pr’ represents precision and ‘Re’ denotes recall.

Method	Bitwidth (W/A)	Blowhole		Break		Crack		Fray		Uneven	
		Pr	Re	Pr	Re	Pr	Re	Pr	Re	Pr	Re
U-Net	32/32	92.4	83.8	96.2	79.9	84.2	74.3	92.4	95.6	63.4	88.8
U-BiNet	1/32	92.6	80.9	95.5	80.9	90.1	70.0	96.7	80.7	78.4	74.1
U-BiNet	1/16	92.9	81.5	92.5	81.2	88.9	71.3	96.3	91.3	74.5	86.5
U-BiNet	1/8	91.1	78.8	93.2	73.6	84.4	75.0	93.1	94.8	76.4	88.9
U-BiNet	1/4	93.6	79.3	96.8	83.0	83.2	70.3	92.7	92.7	73.4	89.6
U-BiNet	1/2	84.2	52.3	90.3	55.0	85.8	35.2	89.1	52.8	68.0	66.6

**Table 4:** Fined-grained defect detection performance on NEU Patches. ‘−’ indicates that the information was not reported or is not known to us.

Method	Bitwidth	Dice	Accuracy	FPR	FNR	MAE	Inference
	(W/A)						Complexity
CAT [49]	-	-	-	0.116	0.030	0.105	-
SSD [50]	-	-	-	0.041	0.677	0.200	-
ESP [11]	-	-	-	0.088	0.266	0.143	-
U-Net [41]	32/32	0.897	0.953	0.013	0.160	0.047	-
U-BiNet	1/32	0.882	0.945	0.008	0.118	0.055	32
U-BiNet	1/8	0.880	0.944	0.012	0.184	0.056	8
U-BiNet	1/4	0.875	0.942	0.012	0.190	0.058	4
U-BiNet	1/2	0.879	0.941	0.013	0.183	0.060	2

**Table 5:** The effects of different components in Bi-ShuffleNet on the final accuracy of the Magnetic Tile dataset.

	Method	TOP-1	OPs
		Accuracy (%)	($\times 10^{7}$)
I	BL	45.51 (±4.01)	2.25
II	BL + ($S_{down}$)	47.96 (±2.04)	2.25
III	BL + ($S_{down}$, $S_{basic}$)	61.23 (±3.71)	2.25
IV	BL + ($S_{down}$, $S_{basic}$, $ks_{DW_{r}}=5$)	68.98 (±3.69)	2.40
V	BL + ($S_{down}$, $S_{basic}$, $ks_{DW_{l},DW_{r}}=5$)	72.04 (±4.00)	2.47
VI	BL + ($S_{down}$, $S_{basic}$, $ks_{DW_{l},DW_{r}}=5$, mp)	74.29 (±2.92)	2.47
VII	BL + ($S_{down}$, $S_{basic}$, $ks_{DW_{l},DW_{r}}=5$, mp, $ks_{conv1}=5$)	75.10 (±3.07)	3.92
VIII	BL + ($S_{down}$, $S_{basic}$, $ks_{DW_{l},DW_{r}}=5$, mp, $ks_{conv1}=9$)	84.29 (±2.63)	8.88
IX	BL + ($S_{down}$, $S_{basic}$, $ks_{DW_{r}}=5$, $2\times ks_{DW_{l}}=3$, mp, $ks_{conv1}=9$)	88.26 (±1.07)	9.01
X	BL + ($S_{down}$, $S_{basic}$, $ks_{DW_{l}}=5$, $2\times ks_{DW_{r}}=3$, mp, $ks_{conv1}=9$)	91.30 (±2.38)	8.99
XI	BL + ($S_{down}$, $S_{basic}$, $2\times ks_{DW_{l},DW_{r}}=3$, mp, $ks_{conv1}=9$)	90.87 (±1.63)	8.86

**Table 6:** Ablation study on NEU.

	Method	TOP-1	OPs
		Accuracy (%)	($\times 10^{7}$)
I	BL	91.11 (±1.07)	1.75
II	BL + ($S_{down}$, $S_{basic}$)	95.56 (±0.70)	1.75
III	BL + ($S_{down}$, $S_{basic}$, $ks_{DW_{r}}=5$)	98.61 (±0.50)	1.90
IV	BL + ($S_{down}$, $S_{basic}$, $ks_{DW_{l},DW_{r}}=5$, mp)	99.56 (±0.22)	1.93
V	BL + ($S_{down}$, $S_{basic}$, $ks_{DW_{l},DW_{r}}=5$, mp, $ks_{conv1}=5$)	99.22 (±0.27)	2.42
VI	BL + ($S_{down}$, $S_{basic}$, $ks_{DW_{l},DW_{r}}=5$, mp, $ks_{conv1}=9$)	99.78 (±0.21)	5.41
VII	BL + ($S_{down}$, $S_{basic}$, $ks_{DW_{r}}=5$, $2\times ks_{DW_{l}}=3$, mp, $ks_{conv1}=9$)	99.28 (±0.67)	4.14
VIII	BL + ($S_{down}$, $S_{basic}$, $ks_{DW_{l}}=5$, $2\times ks_{DW_{r}}=3$, mp, $ks_{conv1}=9$)	99.45 (±0.30)	4.12
IX	BL + ($S_{down}$, $S_{basic}$, $2\times ks_{DW_{l},DW_{r}}=3$, mp, $ks_{conv1}=9$)	99.22 (±0.37)	4.23

**Table 7:** Ablation study on RSiLU. All the activations are full-precision (32-bits).

Dataset	Method	Dice	Acc.	FPR	FNR	MAE
Magnetic Tile	U-BiNet (SiLU)	0.827	0.999	0.242 (×$10^{-3}$)	0.318	0.927 (×$10^{-3}$)
	U-BiNet (RSiLU)	0.814	0.999	0.274 (×$10^{-3}$)	0.260	0.863 ($\times 10^{-3}$)
NEU Patches	U-BiNet (SiLU)	0.874	0.940	0.008	0.202	0.060
	U-BiNet (RSiLU)	0.882	0.954	0.008	0.188	0.055

## Data Availability

Publicly available datasets were analyzed in this study. Magnetic Tile can be found here: https://github.com/abin24/Magnetic-tile-defect-datasets (accessed on 31 August 2021). The NEU dataset can be found here: https://www.kaggle.com/kaustubhdikshit/neu-surface-defect-database?select=NEU-DET (accessed on 15 October 2021).

## Abbreviations

The following abbreviations are used in this manuscript:

CNN	Convolutional neural network
MT	Magnetic Tile
SNR	Signal-to-noise ratio
FNR	False negative rate
FPR	False positive rate
MAE	Mean absolute error
